# Atomistic deformation mechanisms in twinned copper nanospheres

**DOI:** 10.1186/1556-276X-9-335

**Published:** 2014-07-05

**Authors:** Jianjun Bian, Xinrui Niu, Hao Zhang, Gangfeng Wang

**Affiliations:** 1 Department of Engineering Mechanics, SVL, Xi’an Jiaotong University, Xi’an 710049, China; 2 Department of Mechanical and Biomedical Engineering, City University of Hong Kong, 83 Tat Chee Ave, Kowloon, Hong Kong, China; 3 Department of Chemical and Materials Engineering, University of Alberta, Edmonton, AB T6G 2V4, Canada

**Keywords:** Nanosphere, Twin boundary, Strengthening

## Abstract

In the present study, we perform molecular dynamic simulations to investigate the compression response and atomistic deformation mechanisms of twinned nanospheres. The relationship between load and compression depth is calculated for various twin spacing and loading directions. Then, the overall elastic properties and the underlying plastic deformation mechanisms are illuminated. Twin boundaries (TBs) act as obstacles to dislocation motion and lead to strengthening. As the loading direction varies, the plastic deformation transfers from dislocations intersecting with TBs, slipping parallel to TBs, and then to being restrained by TBs. The strengthening of TBs depends strongly on the twin spacing.

## Background

Nanoparticles have been widely used as the reinforced particles in composites, high-performance catalytic and energy harvest materials, etc. [1,2]. Most recently, through advanced fabrication techniques, it is even possible to fabricate nanostructures with controllable internal defects such as twin boundaries (TBs) [3,4]. To explore the wider applications of nanoparticles with TBs, it is imperative to characterize their mechanical properties precisely and understand their fundamental deformation mechanisms.

In nanosized volume, the mechanical behavior depends on not only the intrinsic characteristics such as crystalline structure and internal defects, but also the extrinsic geometry and size. Gerberich et al. measured the hardness of silicon nanospheres with radii in the range of 20 to 50 nm and found that the hardness was up to 50 GPa [5], four times greater than that of bulk silicon. The plastic deformation in silicon nanospheres was theorized to heterogeneous dislocation nucleated at the contact edges and followed by dislocation propagation along a glide cylinder. Molecular dynamic simulations indicated that phase transformation could dominate in silicon nanoparticles [6]. When the diameter of silicon particles was less than 10 nm, dislocation nucleation was suppressed and the hardness lowered with decreasing diameter [7]. Despite the advance in these previous studies, however, the plastic deformation mechanisms in metallic nanoparticles have not yet been fully illuminated. Recently, Bian and Wang revealed that the formation of dislocation lock and deformation twinning dominated in the plastic deformation of copper nanospheres [8].

Coherent twins with low-stacking fault energy could strengthen metals by preventing dislocation from cross-slipping and simultaneously improve ductility by accommodating dislocations gliding parallel to twin planes [4,9]. In addition, TBs could serve as non-regeneration dislocation source contributing to twin migrations [10]. A strengthening-softening transition was exhibited in nanotwinned materials for twin thickness below a critical value, and a discrete twin crystal plasticity model was developed to investigate the size-dependent mechanism [11]. The influence of TBs would be even more prominent in individual small-volume materials. In single crystal nanowires, twin spacing together with sample diameter determined the yield stress [12], and the strengthening resulted from slip arrests at the intersection of partial dislocations and TBs [13]. Twinned copper nanopillars exhibited tension-compression asymmetry, and the plastic deformation could be either reversible or irreversible depending on the stress state. The nucleation and glide of twinning dislocations were the responsible mechanisms for reversible deformation [14], and the subsequent TB migrations could be described by the stick–slip mechanism of coherent TBs [15]. In nanopillars with orthogonally oriented TBs, a brittle-to-ductile transition was observed under uniaxial tension when twin spacing decreased below a critical value. While in nanopillars with slanted TBs, shear offsets and de-twinning dominated the deformation process [3]. For nanoparticles with fivefold twins, TBs greatly increased both the strength and malleability of particles [16]. However, the influence of TBs on the mechanical behavior of metal nanospheres is still unclear up to now. This paper is to investigate the deformation mechanisms in twinned copper nanoparticles subjected to uniaxial compression.

## Methods

**Figure 1:**
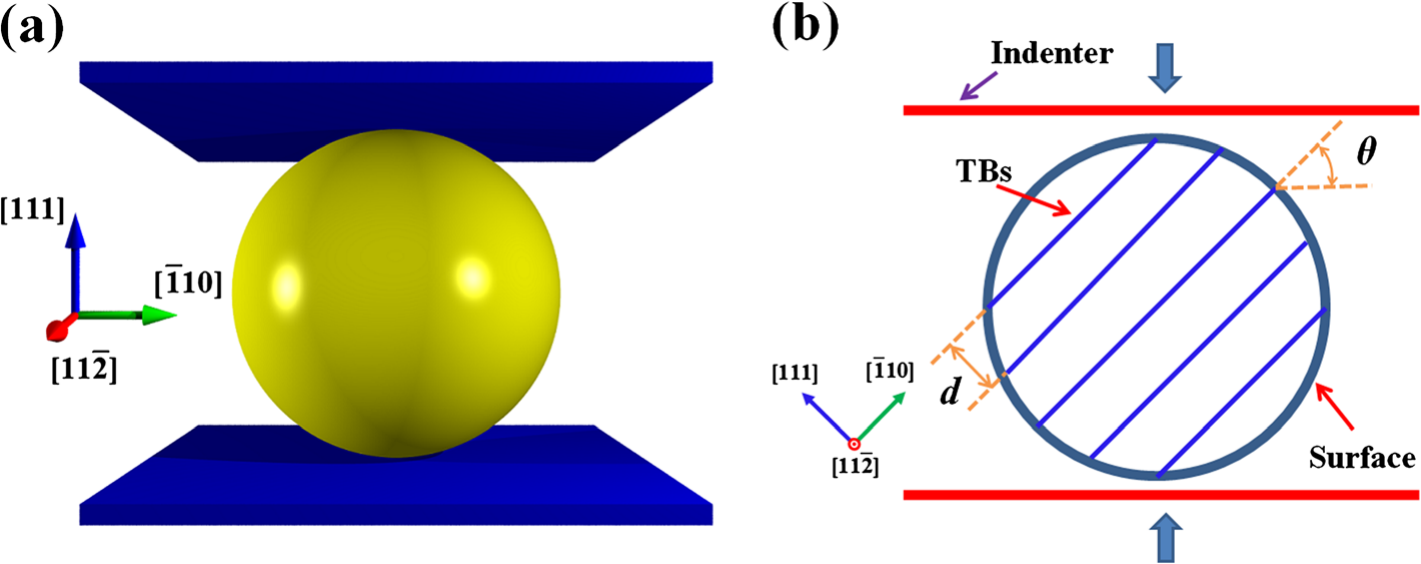
**Schematics of compression of twinned nanospheres.** Simulation model **(a)** and internal twin structures **(b)**.

Consider a face-centered-cubic (fcc) copper nanosphere with parallel (111) coherent TBs under compression, as shown in Figure 1. The twin spacing is *d* and the loading direction varies from [111] to $\left[\bar{1}10\right]$ indicated by a tilt angle *θ* between the twin plane and compressive plane. The embedded atom method (EAM) is utilized to describe the interactions between copper atoms [17], which has been widely adopted for copper crystals [18,19].

To simulate the compression process, a repulsive potential is employed to characterize the interaction between copper atoms and the planar indenter as [20,21]

(1) $$U_{i}\left(\lambda \left(z_{i}-h\right)\right)=K{\left[\lambda \left(z_{i}-h\right)\right]}^{3}H\left(\lambda \left(z_{i}-h\right)\right)$$

where *K* is a specified force constant related to the hardness of indenter, *h* is the position of the compression plane, *λ*(*z*_*i*_ – *h*) is the distance between the *i*-th atom and the planar indenter, *H* is the unit step function, and *λ* equals 1 for the top indenter, −1 for the bottom indenter, respectively.

The molecular dynamics simulations are performed using LAMMPS developed by Sandia National Laboratories. In simulations, the surface of nanosphere is free, except atoms adjacent to the top and bottom indenters experiencing a repulsive potential. An NVT ensemble is chosen with velocity-Verlet integration and a time step of 2.0 fs, and the temperature is controlled at 10 K using a Nosé-Hoover thermostat [22,23]. Before compression, the systems are firstly equilibrated at 10 K for about 20 ps. During compression, the top and bottom indenters simultaneously move toward the center of the sphere with a constant velocity of approximately 10 m/s, and the compression depth *δ* is defined as the decreasing distance between the two indenters.

We fix the radius of nanosphere as 15 nm and investigate the effects of TBs on the deformation of twinned nanoparticle. The total number of atoms in simulations is about 1.2 million. The common neighbor analysis (CNA) method is utilized to analyze the defect structures inside the deformed nanosphere [24]. In this method, atoms in perfect fcc lattice are distinguished from those in hcp lattice, surface, dislocation cores and other defects.

## Results and discussion

**Figure 2:**
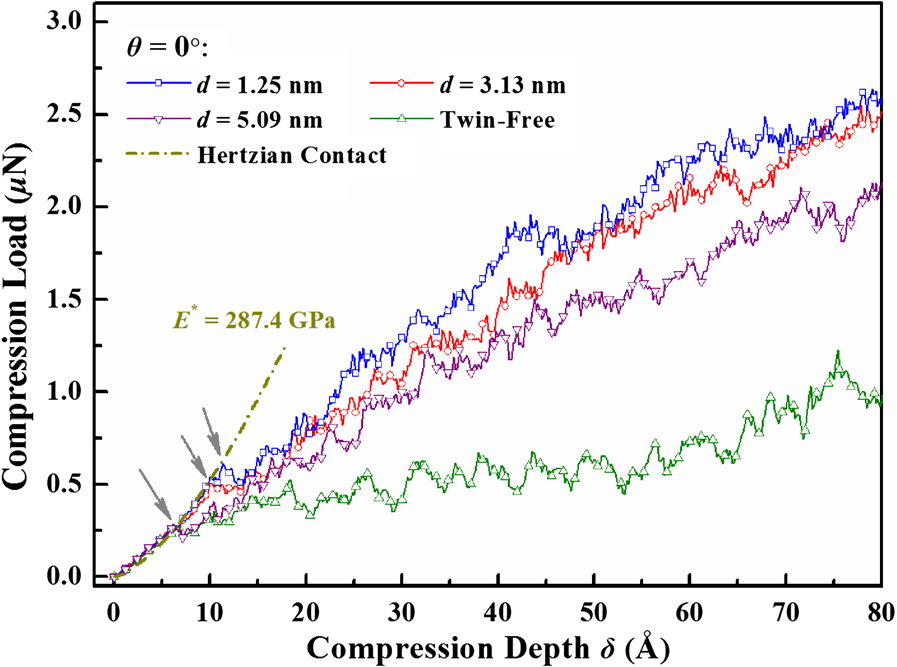
Load versus compression depth response of nanosphere with different twin spacing.

Firstly, we examine the influence of twin spacing in nanosphere with the loading direction perpendicular to the TBs (*θ* = 0°). Figure 2 shows the load response of twinned nanospheres with twin spacing *d* varying from 1.25 to 5.09 nm. For comparison, the load response of a twin-free nanosphere is also included.

When measuring the elastic properties of single crystal materials, the classical Hertzian contact theory is widely used to estimate the reduced modulus in MD simulations [6,25]. For the compression of an elastic sphere with radius of *R*, Hertzian theory predicts the relationship between applied load *F* and compression depth *δ* as [26]

(2) $$F=\frac{4}{3}E^{*}R^{1/2}{\left(\frac{\delta }{2}\right)}^{3/2}$$

where E^*^ is the reduced Young’s modulus of the sphere. In this paper, E^*^ is fitted from the load versus compression depth relation in the elastic regime by Equation 2. For different twin spacing, the value of E^*^ keeps almost the same as 287.4 GPa. It is seen that the elastic response of nanosphere under compression is determined mainly by the local elastic properties under indenter. Therefore, for a given loading direction, the change of twin spacing does not affect the overall elastic response of nanosphere. And the reduced modulus is much larger than the theoretical prediction 153 GPa of the bulk single crystal material in <111 > direction [27]. In nanowires and nanoparticles, improved elastic modulus and yield stress have also been observed [5,13].

**Figure 3:**
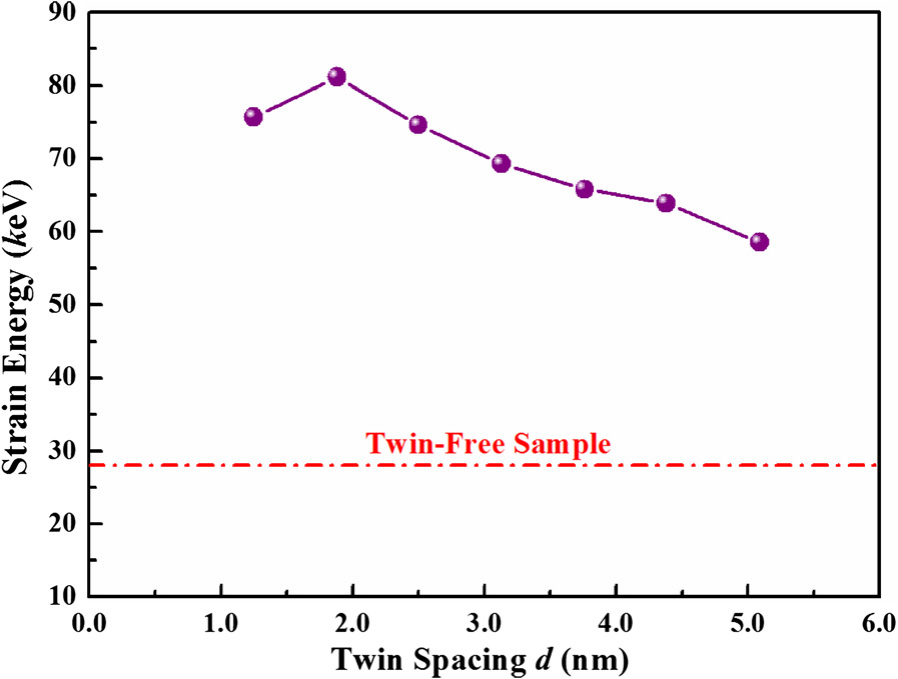
**Strain energy of the deformed nanosphere as a function of twin spacing up to *δ*/*R*** **= 53.3%.**

However, the introduction of TBs plays an important role in plastic deformation. The first load-drop, as marked by arrows in Figure 2, indicates the appearance of initial yield. The local peak load corresponding to the first load-drop may be considered as the yield load. It is found that, when the twin spacing decreases from 5.09 to 1.25 nm, the yield load increases from 0.28 to 0.62 μN. In the further development of plasticity, the compression load of the twinned nanosphere is significantly larger than that of the twin-free nanosphere for the same compression depth. The highly serrated load-compression response is indicative of dislocation activities inside the deformed nanospheres. To estimate the influence of TBs qualitatively, the strain energy stored in nanospheres up to a given compression depth (*δ*/*R* = 53.3%) is also shown in Figure 3. It is found that, the strain energy of twinned nanospheres increases clearly as the twin spacing decreases, reaching its maximum at the twin spacing of 1.88 nm, and then declines with further decreasing twin spacing. Such characteristics are similar to those in nanotwinned polycrystalline materials [4,9].

**Figure 4:**
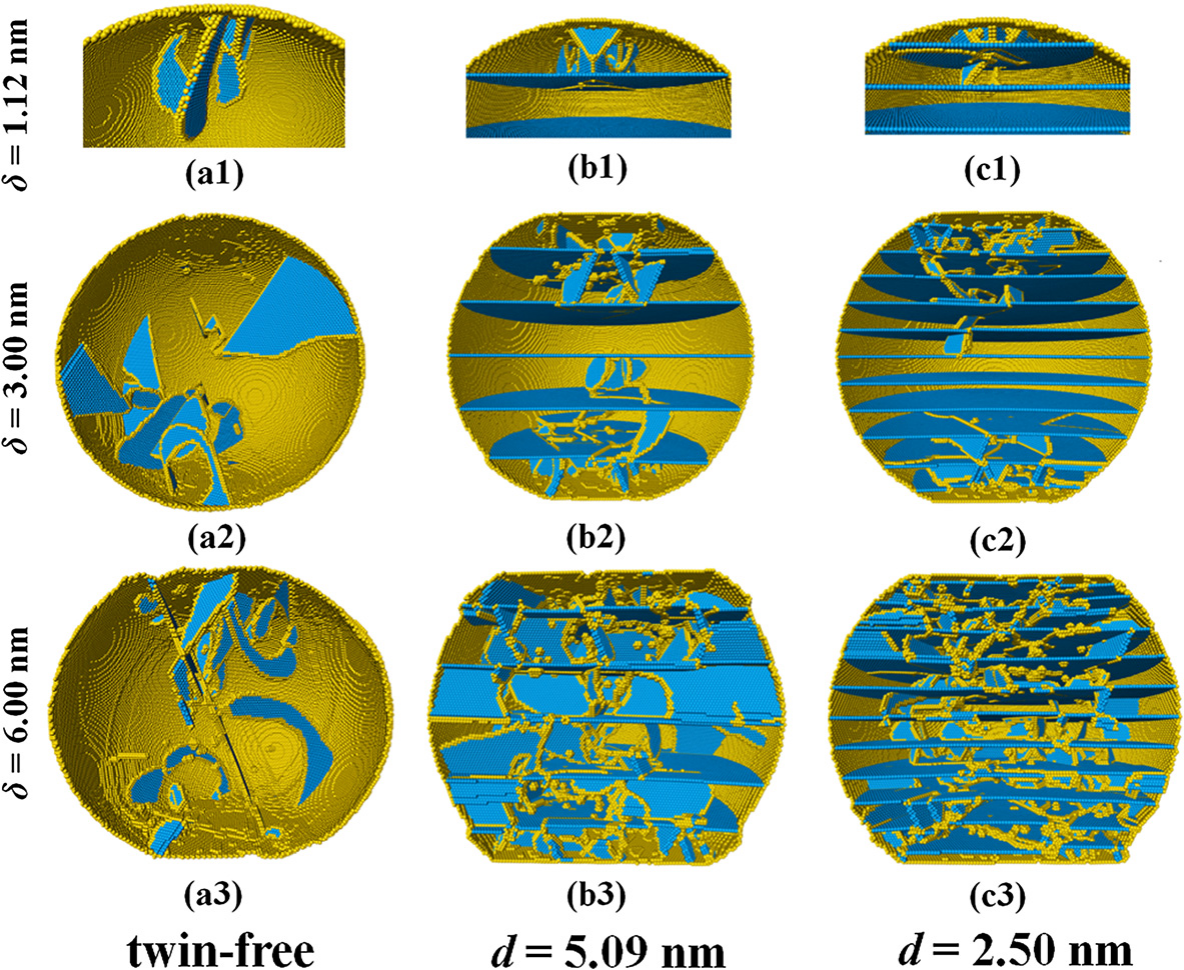
**Atomic defect structures inside nanosphere with different twin spacing.** Atoms are colored by their CNA parameters, and those in perfect fcc lattice are not shown. Coloring scheme: yellow for atoms at surface, dislocation cores, or other defects and blue for atoms in TBs or stacking faults.

In order to understand the underlying strengthening mechanisms, we examine the atomistic structures in plastic stage for several samples, as shown in Figure 4. For a twin-free nanosphere, the plastic deformation begins with the nucleation of partial dislocations from the contact edge, and the dislocations then glide on {111} slip planes. Without experiencing obstacles from TBs, most partial dislocations easily glide to the opposite surface and annihilate here, forming surface steps. This process exhausts nucleated dislocations in nanosphere and reduces dislocation density, corresponding to the dislocation starvation mechanism. In the compression of twinned nanospheres, dislocation embryos will still nucleate from the contact fringe. However, the existence of TBs hinders dislocation gliding, and the volume between the initial contact surface and the topmost TB determines when the first load-drop occurs, similar to that observed in nanocrystallines [28]. When the volume is large, there is ample space for dislocation gliding, the first load-drop is close to that of the twin-free sample, i.e., *d* = 5.09 nm. When the volume is small, dislocations are hindered after impinging the TB, and the cutting through TB results in the first load-drop. The smaller the volume, the larger the yield load.

When the compression direction is perpendicular to TBs, the slip directions and slip planes of most dislocations are intersecting with twin planes. With the compression increasing and plastic deformation developing toward the center of nanospheres, dislocations will have to cut through TBs one by one, which corresponds to the strengthening of dislocation-TB interaction [29,30]. Another main strengthening in twinned nanospheres comes from the formation of Lomer dislocations. As an extended dislocation is driven into a coherent TB by progressive compression, it recombines into a perfect dislocation at the coherent TB. After slipping through the TB, instead of splitting into Shockley partials, many full dislocations glide on {100} planes in next twin lamella and form {100} < 110 > Lomer dislocations.

When the twin spacing is large, there is ample room in twin lamella for Lomer dislocation cross-slip and dissociation. A Lomer dislocation firstly cuts through new TBs after reaching them, then cross-slips on to the usual {111} slip plane and dissociates into two partial dislocations, connected by a stacking fault. While the remaining dislocation segments in the original twin lamella rotate to form pure screw Lomer dislocation segments, then they also cross-slip on to {111} planes and dissociate into extended dislocations. In subsequent deformation, both the extended dislocations in original and new twin lamellas will form new Lomer dislocations after reaching TBs. These repeated cross-slips and dissociations of Lomer dislocations generate complex dislocation network inside nanospheres [31].

When the twin spacing is smaller than a critical value (such as *d* < 1.88 nm), there is no ample room between TBs, and dislocation dissociation is highly restricted. This is different from that in bulk nanotwinned material with small twin spacing when both cross-slip and dissociation are suppressed [31]. The jogged full dislocation could quickly cut through TBs after generation, passing the central region of nanosphere. This process leaves a large number of partial dislocations at twin planes. Plasticity accommodated by the gliding of these twinning dislocations is a softening mechanism in nanotwinned materials [9,30]. In addition, the restriction of small twin spacing on dislocation dissociation also decreases the obstacles for the subsequent glide of dislocations in twin lamellas.

**Figure 5:**
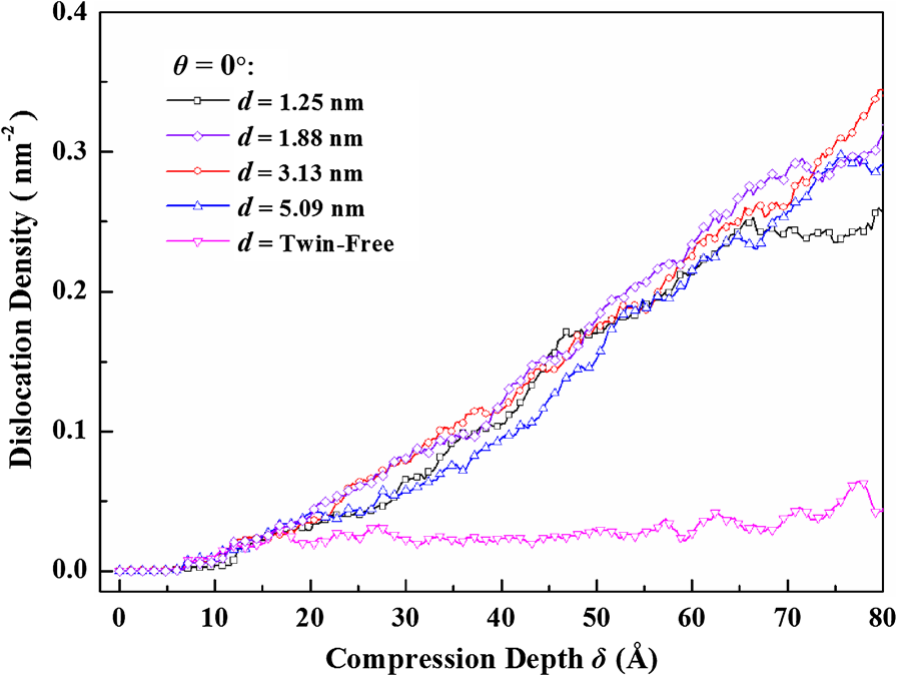
Evolution of dislocation density inside nanosphere with different twin spacing.

The dislocation density is also an indicator of plastic deformation. The evolutions of dislocation densities versus compression depth are depicted in Figure 5. It is noted that for the compression of the twin-free nanosphere, the dislocation density maintains nearly a constant for *δ*/*R* > 13.3% when the nucleation of dislocations is balanced by the dislocation exhaustion. While for the twinned nanospheres, the dislocation density increases gradually as compression progresses. Decreased twin spacing increases dislocation density, while continuous refinement of twin spacing below 1.88 nm does not improve dislocation density apparently. We also use the newer potential developed by Mishin et al. [32] to simulate the same problem, and quite similar deformation characteristics are observed.

**Figure 6:**
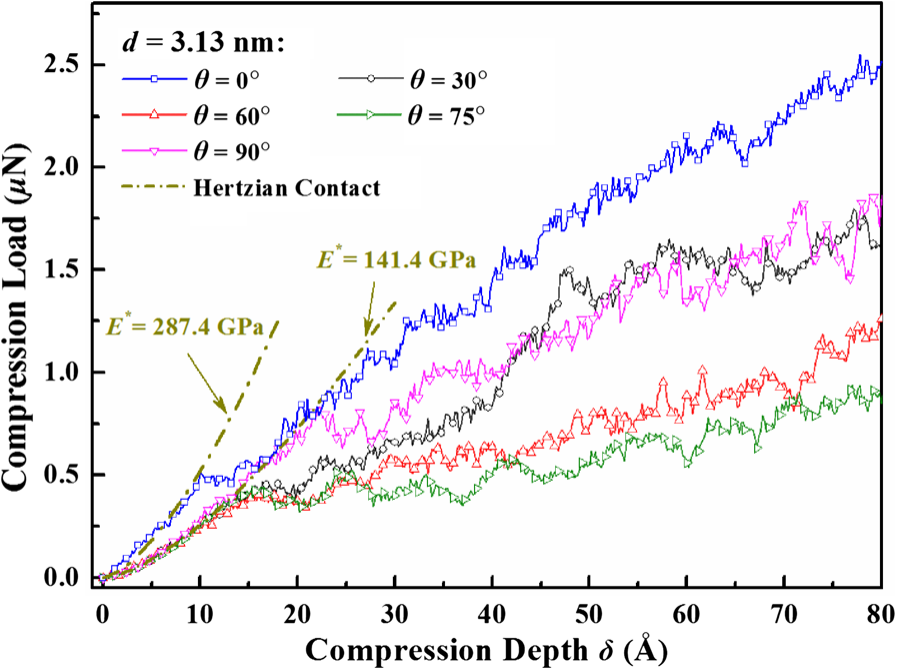
Load versus compression depth response of nanosphere with different twin tilt angle.

Then we examine the influence of loading direction by fixing the TB spacing at 3.13 nm and changing the tilt angle *θ* from 0° to 90°. Figure 6 gives the corresponding load-compression depth relation. The reduced Young’s modulus in different loading directions is fitted by the Hertzian contact theory (Equation 2). Owing to the local mechanical property under indenter varies as the loading direction changes, the reduced Young’s modulus declines quickly from 287.4 to 141.4 GPa. As shown in Figure 6, when the twin tilt angle *θ* is larger than 10°, the averaged atom compactness in compression direction is close to that in <110 > direction; hence, all the fitted reduced elastic moduli are around 141.4 GPa, which is close to the theoretical prediction 148.7 GPa of bulk material in <110 > direction [27].

**Figure 7:**
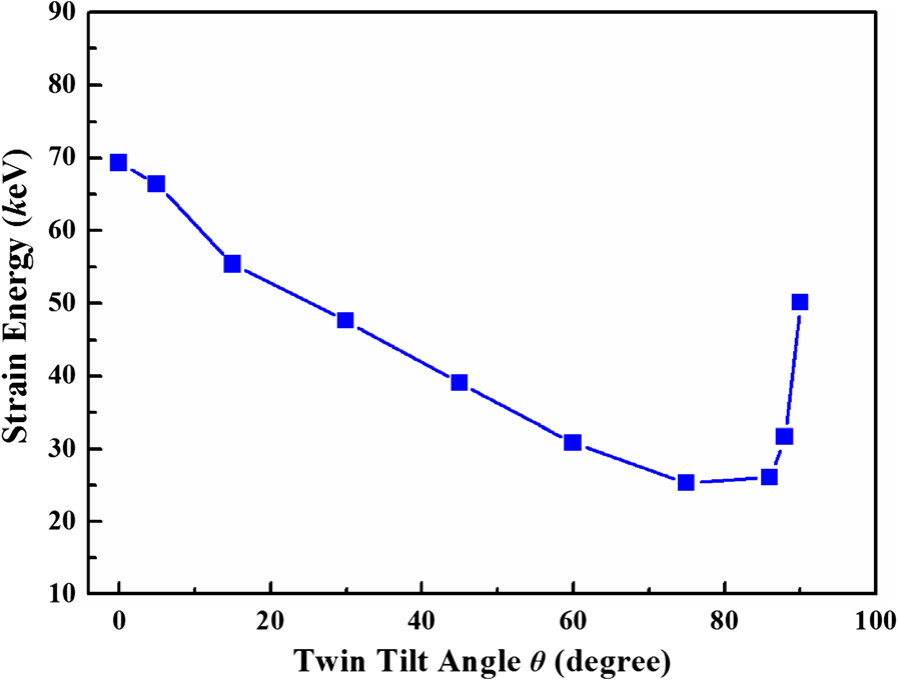
**Strain energy of the deformed nanosphere as a function of twin tilt angle up to *δ*/*R*** **= 53.3%.**

In the plastic deformation regime, the load-compression depth curves tend to decline continuously as the tilt angle *θ* increases from 0° to 75°, while rise as the tilt angle *θ* increases further from 75° to 90°. Such dependence on loading direction also appears in the strain energy up to a given compression *δ*/*R* = 53.3%, as displayed in Figure 7. The variation of plastic deformation in different loading direction implies a possible switch of deformation mechanism in nanospheres.

**Figure 8:**
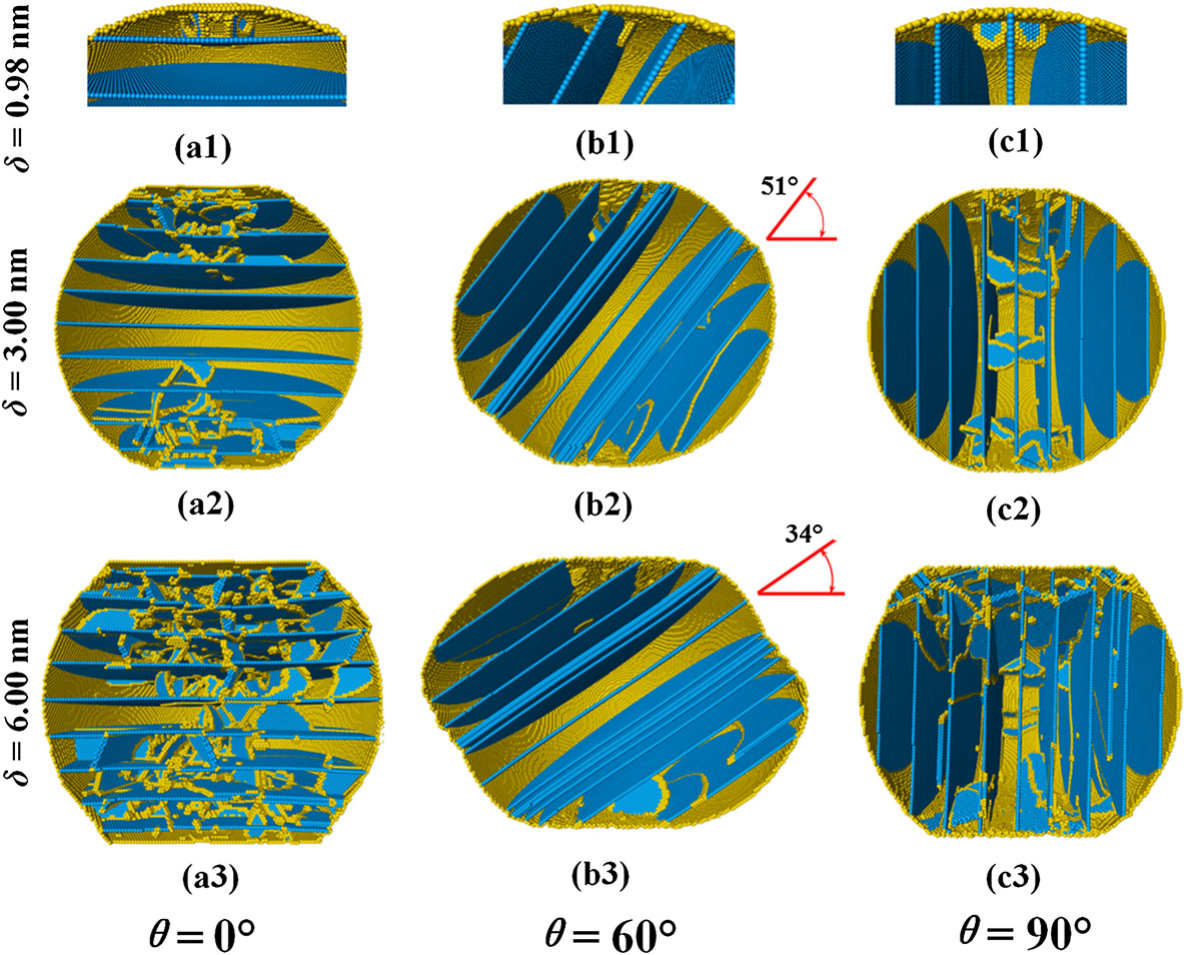
**Atomic defect structures inside twinned nanosphere under different loading direction.** The identification and coloring scheme of atoms are the same as that of Figure 4.

**Figure 9:**
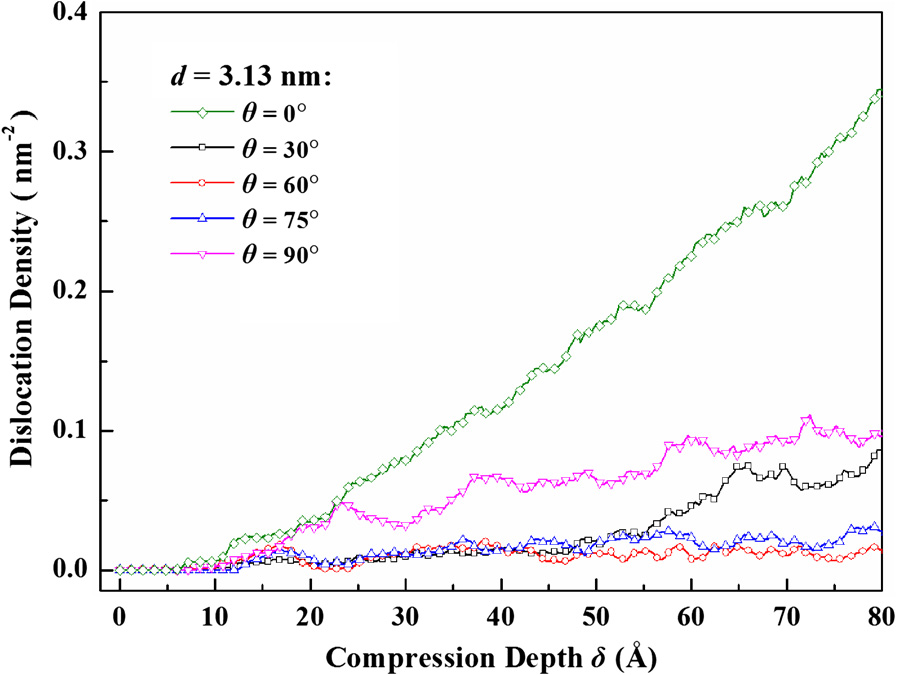
Evolution of dislocation density inside nanosphere with different twin tilt angle.

Figure 8 examines the atomic patterns inside three nanospheres with various loading directions. In all cases, dislocations will nucleate from the contact fringes, as shown in a1, b1, and c1 of Figure 8. As the tilt angle *θ* increases from 0° to 75°, the motion of dislocations will transfer from intersecting with twin planes to slipping parallel to the twin planes, and thereby the blocking effect of TBs will decrease [29,30]. The slip of dislocations results in the migration of TBs or the generation of stacking faults spanning twin lamellae, as shown in b2 of Figure 8. It is also interesting to notice that TBs tend to rotate toward the compression plane, as shown in b2 and b3 of Figure 8. When the tilt angle *θ* is close to 90°, though the glide direction of dislocations is parallel to TBs, the slip planes are inclined to the twin planes. Both the leading and trailing partials, connected by stacking fault ribbons, are bounded by neighboring TBs while expanding as shown in c2 and c3 of Figure 8, which lead to another strengthening mechanism of twin-dislocation interactions [29,30]. The corresponding dislocation density evolution is depicted in Figure 9. It is noted that when the twin tile angle *θ* is equal to 0° or 90°, the resultant dislocation density is apparently larger than those in other cases.

## Conclusions

In the present study, MD simulations are performed to address the influence of TBs on the compression of nanospheres. The elastic response of twinned nanospheres under compression is determined mainly by the local elastic properties under indenter and still can be captured by the classical Hertzian contact model. Compared to the twin-free sample, the existence of TBs in nanospheres greatly increases the strain hardening in plastic deformation, depending on the twin spacing and loading direction. As the tilt angle between compression plane and TBs increases from 0° to 75°, the strengthening of TBs declines, while increases again as the tilt angle approaches to 90°. Correspondingly, the plastic deformation mechanism switches from intersecting with TBs, slipping parallel to TBs, and then to being restrained by TBs, as the tilt angle increases. Moreover, the enhancement of TBs increases evidently as the twin spacing decreases, obtaining its maximum at a critical twin spacing, and then declines.

## Competing interests

The authors declare that they have no competing interests.

## Authors’ contributions

JB conducted the MD simulations. GW designed the project. JB and GW drafted the manuscript. XN and HZ revised the paper. All authors read and approved the final manuscript.
